# Does the association of the triglyceride to high-density lipoprotein cholesterol ratio with fasting serum insulin differ by race/ethnicity?

**DOI:** 10.1186/1475-2840-7-4

**Published:** 2008-02-28

**Authors:** Chaoyang Li, Earl S Ford, Yuan-Xiang Meng, Ali H Mokdad, Gerald M Reaven

**Affiliations:** 1 National Center for Chronic Disease Prevention and Health Promotion, Centers for Disease Control and Prevention, Atlanta, Georgia, USA; 2 Department of Family Medicine, Morehouse School of Medicine, Atlanta, Georgia, USA; 3 Department of Medicine, Stanford University School of Medicine, Stanford, California, USA

## Abstract

**Background:**

The triglyceride to high-density lipoprotein cholesterol (TG/HDL-C) ratio has been reported to be as closely correlated with insulin resistance as is the fasting serum insulin concentration (FSI), and therefore it is seen as a clinically useful way to identify the concomitant presence of insulin resistance and dyslipidemia. However, conflicting findings exist for the association of the TG/HDL-C ratio with FSI by race/ethnicity.

**Methods:**

The associations of FSI concentration, serum triglyceride concentrations, and HDL-C were analyzed using log-binomial regression analyses and receiver operating characteristic (ROC) curve analysis among nondiabetic adults (n = 2652, aged ≥ 20 years, 51.2% men) in the United States.

**Results:**

After adjustment for potential confounding effects, the prevalence ratio of hyperinsulinemia was 2.16 (95% confidence interval [CI], 1.74 to 2.08) when using a single cutoff point of 3.5, and 2.23 (95% CI, 1.83 to 2.72) when using race/ethnicity-specific cutoff points of 3.0 for non-Hispanic whites and Mexican Americans and 2.0 for non-Hispanic blacks for the TG/HDL-C ratio. The area under the ROC curve of the TG/HDL-C ratio for predicting hyperinsulinemia was 0.77 (95% CI, 0.74 to 0.79), 0.75 (95% CI, 0.69 to 0.77), and 0.74 (95% CI, 0.69 to 0.76) for non-Hispanic whites, non-Hispanic blacks, and Mexican Americans, respectively.

**Conclusion:**

There was a significant association between the TG/HDL-C ratio and FSI among three major racial/ethnic groups in the United States. Our results add further support to the notion that the TG/HDL-C ratio may be a clinically simple and useful indicator for hyperinsulinemia among nondiabetic adults regardless of race/ethnicity.

## Background

The prevalence of hyperinsulinemia has increased by about 35% among nondiabetic adults in the past decade in the United States [1]. Insulin resistance has been proposed as an underlying cause of type 2 diabetes and the metabolic syndrome [2,3] and has been found to be associated with increased risk for cardiovascular diseases [2,4,5]. The availability of relatively simple measures to identify apparently healthy people who are sufficiently insulin resistant to be at increased risk of developing type 2 diabetes, cardiovascular disease, and the numerous other clinical syndromes that occur with increased frequency in insulin-resistant persons would be of significant clinical benefit [6-8].

The triglyceride to high-density lipoprotein cholesterol (TG/HDL-C) concentration ratio has been reported to be as closely related to insulin resistance as is the fasting plasma insulin concentration [9,10], a commonly used surrogate estimate of insulin resistance. Previous studies have demonstrated that fasting insulin was highly correlated with homeostasis model assessment (HOMA) and quantitative insulin sensitivity check index (QUICKI) (correlation coefficients >0.95) of insulin resistance among nondiabetic individuals [11-13]. In contrast to measures of lipid and lipoprotein concentrations, there has been no attempt to standardize assays of insulin concentration to date. Thus, it seemed possible that use of the TG/HDL-C ratio, based on commonly available and standardized measurements, could help clinicians identify persons who were not only insulin resistant but also displayed the characteristic dyslipidemia of people with this defect in insulin action [9,10]. The relationship between the TG/HDL-C ratio and a direct measure of insulin resistance was first reported among 258 overweight or obese adults, of whom 87% were non-Hispanic whites [9]. This association has been replicated in a larger clinically based sample that represented the general population [10]. Elsewhere, in a study in an East African population, the TG/HDL-C ratio was found to be significantly associated with insulin resistance as measured by HOMA [14]. In contrast, recent studies have reported that the triglyceride or triglycerides/HDL-C ratio was not significantly associated with insulin resistance in black adults [15] and adolescents [16].

In previous studies, black males have had lower triglyceride concentrations and higher HDL-C concentrations than white males, while versus white females, black females have had similar or lower triglyceride concentrations and comparable HDL-C concentrations [17]. Elsewhere, Mexican American males were found to have higher fasting serum insulin (FSI) concentrations than white or black males, while adult black or Mexican American females were reported to have higher FSI concentrations than their white counterparts [18]. In US youth aged 12–17 years, there were no significant variations by race/ethnicity among males, while black females had higher FSI concentrations than white or Mexican American females [19].

Because of racial/ethnic variations in triglyceride, HDL-C, and FSI concentrations, the association of the TG/HDL-C ratio with FSI may differ by race/ethnicity. To the best of our knowledge, no studies have investigated the possibility of such differences. Thus, the goal of this study was to use a nationally representative sample to determine whether the association of triglyceride, HDL-C, and the TG/HDL-C ratio with the FSI may differ between non-Hispanic whites, non-Hispanic blacks, and Mexican Americans.

## Methods

### Study design and participants

In the National Health and Nutrition Examination Survey (NHANES) conducted during 1999–2002, the samples were recruited using a multistage, stratified sampling design to represent the noninstitutionalized civilian US population. After being interviewed at home, participants were invited to attend the mobile examination center, where they provided a blood sample and were examined. Details about the survey may be found elsewhere [20,21].

We limited the analyses to men and nonpregnant women aged ≥ 20 years who attended the morning medical examination and had fasted ≥ 8 hours. All participants who had a positive history of diabetes or currently had a fasting glucose concentration ≥ 126 mg/dL (7.0 mmol/L) were excluded. Participants of other races and ethnicities were excluded because of small samples. The final sample (n = 2652; unweighted: 51.2% men, 56.8% non-Hispanic whites, 18.1% non-Hispanic blacks, and 25.1% Mexican Americans; weighted: 49.4% men, 82.2% non-Hispanic whites, 10.5% non-Hispanic blacks, and 7.3% Mexican Americans) represents nondiabetic adults aged ≥ 20 years in the United States.

### Procedures

Serum specimens were frozen at <-70°C, shipped on dry ice, and stored at <-70°C until analysis. All insulin assays were performed at the same laboratory of the University of Missouri at Columbia. FSI concentration was measured using a radioimmunoassay kit from Pharmacia Diagnostics AB (Uppsala, Sweden). The cross-reactivity of Pharmacia insulin antibody with proinsulin is approximately 40%. Quality control procedures followed modified Westgard rules and included both within- and between-assay quality control procedures using Levy-Jennings chart plots for means and ranges with monitoring of trend. The overall coefficients of variation ranged from 3.3% to 5.4% in NHANES 1999–2002. Details of the laboratory procedures for insulin assay are found elsewhere [20,21]. Plasma glucose concentration was measured using an enzymatic reaction. Serum triglyceride concentrations were measured enzymatically after hydrolyzation to glycerol. HDL-C was measured after the precipitation of other lipoproteins with a heparin-manganese chloride mixture. C-reactive protein (CRP) concentrations were quantified by latex-enhanced nephelometry (N High Sensitivity CRP assay) on a BN II nephelometer (Dade Behring Inc., Deerfield IL).

Waist circumference was measured to the nearest 0.1 cm with a steel measuring tape at the high point of the iliac crest at minimal respiration. Body mass index [BMI = weight (kg)/height (m)^2^] was calculated using measured weight and height, and BMIs were categorized into three groups (1: <25, 2: 25 to 29.9, and 3: ≥ 30) according to World Health Organization criteria [22]. Up to four blood pressure readings were obtained in the mobile examination center; for participants with three or four the average of the last two was used to establish blood pressure status; if there were only two measurements, the second one was used (a few participants had just one measurement).

### Statistical analyses

We assessed the distribution and normality of continuous variables and performed logarithmic transformation for the FSI, triglycerides, HDL-C, and the triglycerides/HDL-C ratio to approximate a normal distribution. Multiple linear regression models were performed to assess the association of triglycerides, HDL-C, and the triglycerides/HDL-C ratio with FSI while adjusting for potential confounders including age, education, poverty-income ratio, smoking status, systolic blood pressure, CRP, and waist circumference. Standardized regression coefficients were estimated to facilitate comparisons across variables with different metric scales.

We defined hyperinsulinemia using the 75^th ^percentile cutoff values of the FSI concentration among nondiabetic participants in NHANES 1999–2002 according to the suggestion of the European Group for the Study of Insulin Resistance [23]. The area under the receiver-operating characteristic (ROC) curve was used to examine the predictive value of triglyceride, HDL-C, and the triglycerides/HDL-C ratio for hyperinsulinemia by race/ethnicity separately. Values for the area under the ROC curve of 0.5, ≥ 0.7 but < 0.8, ≥ 0.8 but < 0.9, and ≥ 0.9 have been suggested as reflecting the following levels of discrimination: none, acceptable, excellent, and outstanding [24]. The Youden index was calculated using the following formula: sensitivity + specificity – 1 [25], and the maximum value of the Youden index corresponded to the optimal cutoff point [26]. The areas under the ROC curve were estimated and compared between racial/ethnic subgroups using the SAS macros that were created specifically to account for the sampling weights of survey data [27]. In addition, we estimated the prevalence of hyperinsulinemia associated with the proposed TG/HDL-C ratio ≥ 3.5 [10] stratified by BMI and race/ethnicity. The prevalence ratio (PR) and 95% confidence interval (CI) were estimated using the log-binomial regression analysis [28]. An α of 0.05 was used as a statistical significance level for two-sided tests. All analyses were conducted using SAS (version 9.1) and SUDAAN software (Release 9.0, Research Triangle Institute, Research Triangle Park, NC) to account for the complex sampling design.

## Results

**Figure 1:**
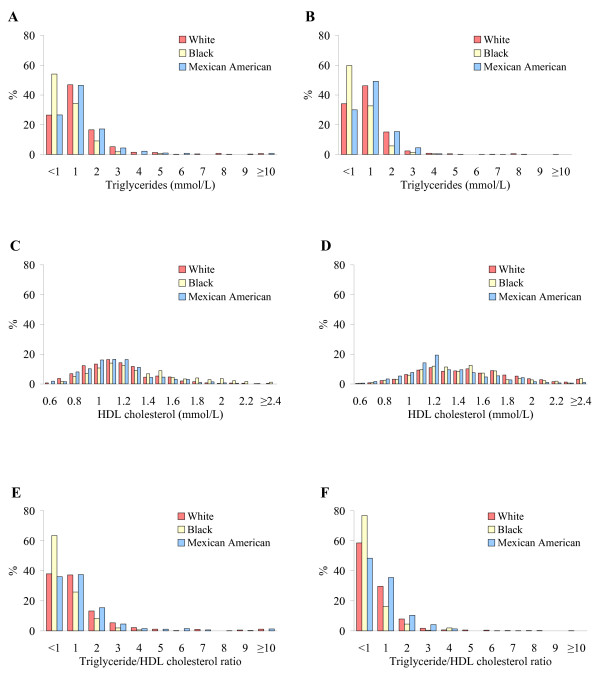
Distribution of triglycerides, HDL-C, and the TG/HDL-C ratio in original metric scale by sex and race/ethnicity. A, triglycerides in men; B, triglycerides in women; C, HDL-C in men; D, HDL-C in women; E, the TG/HDL-C ratio in men; and F, the TG/HDL-C ratio in women.

The distribution of triglycerides, HDL-C, and TG/HDL-C ratio appeared to be similar between men and women, but different between racial/ethnic groups (Figure 1). Pearson correlation coefficients were -0.41, 0.93, and -0.71 for triglycerides versus HDL-C, triglycerides versus the TG/HDL-C ratio, and HDL-C versus the TG/HDL-C ratio, respectively.

Among men, non-Hispanic blacks had a lower mean triglyceride concentration, a higher mean HDL-C concentration, and a lower TG/HDL-C ratio than both non-Hispanic whites and Mexican Americans (Table 1). In contrast, Mexican American men had a higher FSI concentration than their non-Hispanic white and non-Hispanic black counterparts. Among women, non-Hispanic blacks had a lower mean triglyceride concentration and lower TG/HDL-C ratio than both non-Hispanic whites and Mexican Americans. Mexican Americans, in contrast, had a lower mean HDL-C concentration than both non-Hispanic whites and non-Hispanic blacks. Non-Hispanic blacks and Mexican Americans had a higher mean FSI than non-Hispanic whites.

**Table 1:** Geometric means of triglycerides, HDL-C, the TG/HDL-C ratio, and FSI by sex and race/ethnicity

	Non-Hispanic White (NHW)			Non-Hispanic Black (NHB)			Mexican American (MA)			*P *value†		
				
	Mean*	95% CI		Mean	95% CI		Mean	95% CI		NHB vs. NHW	MA vs. NHW	MA vs. NHB
**Men**	N = 770			N = 243			N = 346					
Triglycerides (mmol/L)‡	1.46	1.38	1.54	1.03	0.95	1.12	1.46	1.31	1.63	<0.001	0.975	<0.001
HDL-C (mmol/L)§	1.13	1.11	1.16	1.28	1.24	1.33	1.12	1.09	1.15	<0.001	0.588	<0.001
TG/HDL-C ratio	1.29	1.20	1.38	0.81	0.73	0.90	1.30	1.14	1.49	<0.001	0.863	<0.001
FSI (pmol/L)||	58.0	55.3	60.9	56.4	52.4	60.7	69.4	65.1	73.8	0.494	<0.001	<0.001
												
**Women**	N = 737			N = 236			N = 320					
Triglycerides (mmol/L)	1.27	1.23	1.32	0.94	0.88	1.00	1.30	1.21	1.40	<0.001	0.671	<0.001
HDL-C (mmol/L)	1.43	1.39	1.46	1.39	1.34	1.44	1.28	1.24	1.31	0.287	<0.001	<0.001
TG/HDL-C ratio	0.89	0.85	0.94	0.67	0.62	0.73	1.02	0.93	1.11	<0.001	0.030	<0.001
FSI (pmol/L)	51.1	48.9	53.4	67.8	62.7	73.3	67.9	61.6	74.9	<0.001	<0.001	0.966

HDL = high-density lipoprotein; FSI = fasting serum insulin; CI = confidence interval. * Weighted unadjusted geometric means.

† P-values indicate the significance of the differences in the means of triglycerides, HDL-C, the TG/HDL-C ratio, and FSI between two racial/ethnic groups. NHW = non-Hispanic white, NHB = non-Hispanic black, MA = Mexican American. Significance level with Bonferroni adjustment for multiple comparisons is 0.05/3 ≈ 0.017.

‡ To convert triglyceride concentration to mg/dl, divide concentration in mmol/L by 0.01129.

§To convert HDL-C concentration to mg/dl, divide concentration in mmol/L by 0.02586.

|| To convert FSI concentration to μU/mL, divide concentration in pmol/L by 6.

After adjustment for potential confounding effects, triglycerides, HDL-C, and the TG/HDL-C ratio were significantly associated with FSI in the three racial/ethnic groups (Table 2). No significant difference in the regression coefficients between any two racial/ethnic groups was detected (all *P *> 0.017 with Bonferroni adjustment). There was no significant interaction between race/ethnicity and triglycerides, HDL-C, or the TG/HDL-C ratio (*P *ranged from 0.22 to 0.98) on the FSI in the combined data.

**Table 2:** Metric and standardized regression coefficients of triglycerides, HDL-C, and the TG/HDL-C ratio on FSI by sex and race/ethnicity

	Non-Hispanic White (NHW)			Non-Hispanic Black (NHB)			Mexican American (MA)			*P*-value‡			
				
	β_m_*	SE	β_s_†	β_m_	SE	β_s_	β_m_	SE	β_s_	NHB vs. NHW	MA vs. NHW	MA vs. NHB	Interaction§
**Men**	n = 770			n = 243			n = 346						
Triglycerides (mmol/L)	0.22	0.03	0.25	0.30	0.06	0.23	0.28	0.06	0.31	0.13	0.63	0.29	0.22
HDL-C (mmol/L)	-0.51	0.07	-0.24	-0.45	0.12	-0.20	-0.46	0.10	-0.21	0.98	0.88	0.94	0.98
TG/HDL-C ratio	0.19	0.02	0.27	0.24	0.04	0.25	0.22	0.04	0.31	0.14	0.75	0.32	0.27
													
**Women**	n = 737			n = 236			n = 320						
Triglycerides (mmol/L)	0.26	0.07	0.26	0.28	0.08	0.24	0.44	0.05	0.42	0.67	0.03	0.05	0.23
HDL-C (mmol/L)	-0.46	0.07	-0.25	-0.32	0.11	-0.18	-0.61	0.12	-0.28	0.72	0.31	0.26	0.60
TG/HDL-C ratio	0.24	0.05	0.30	0.21	0.05	0.24	0.34	0.03	0.40	0.55	0.05	0.05	0.26

HDL = high-density lipoprotein; FSI = fasting serum insulin. W = non-Hispanic white, B = non-Hispanic black, M = Mexican American.

* β_m _– metric regression coefficient. SE = standard error.

† β_s _– standardized regression coefficient. β_m _and β_s _were adjusted for age, education attainment, poverty-income ratio, smoking, systolic blood pressure, C-reactive protein, and waist circumference. All individual regression coefficients were significant at α = 0.05 level.

‡ Significance level with Bonferroni adjustment for multiple comparisons is 0.05/3 ≈ 0.017.

§Interaction between race/ethnicity and TG/HDL-C ratio.

**Figure 2:**
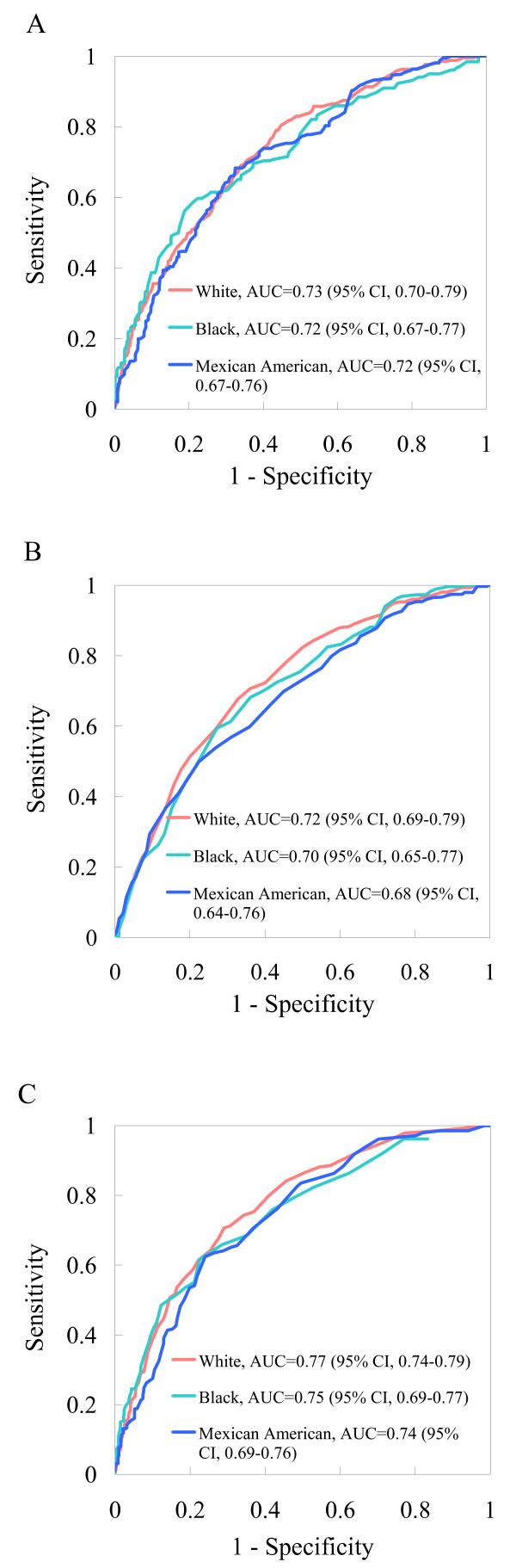
ROC curve of triglycerides (A), HDL-C (B), and the TG/HDL-C ratio (C) for the prediction of hyperinsulinemia by race/ethnicity. The 75^th ^percentile cutoff value of fasting insulin was 13.13 uU/mL. AUC = area under curve.

The 75^th ^percentile cutoff point for FSI was 78.77 pmol/L (13.131 uU/mL) among nondiabetic people in NHANES 1999–2002. The area under the ROC curve of the TG/HDL-C ratio (AUC = 0.75; 95% CI, 0.73–0.78) was larger than that of triglyceride (AUC = 0.72; 95% CI, 0.69–0.74; *P *< 0.0001) or HDL-C (AUC = 0.72; 95% CI, 0.69–0.74; *P *= 0.0007) alone for the prediction of hyperinsulinemia (Figure 2). According to the maximum value of the Youden index, the optimal cutoff point for the TG/HDL-C ratio was 1.2 in mmol/L unit (or equivalent to 3 in mg/dl unit) for both non-Hispanic whites and Mexican Americans and 0.9 in mmol/L unit (or equivalent to 2 in mg/dl unit) for non-Hispanic blacks. The corresponding sensitivity and specificity were 70.6% and 71.0% in non-Hispanic whites, 61.6% and 77.4% in non-Hispanic blacks, and 64.1% and 71.1% in Mexican Americans.

The unadjusted prevalence of hyperinsulinemia was more than doubled among people with TG/HDL-C ≥ 3.5 (or the new cutoff point) than those with TG/HDL-C < 3.5 (or the new cutoff point); and this pattern was consistent across the three BMI categories and the three racial/ethnic groups (Table 3). Using the cutoff points of 3.0 for non-Hispanic white and Mexican Americans and 2.0 for non-Hispanic blacks yielded lower prevalence rates of hyperinsulinemia, but larger prevalence ratios compared to the cutoff point of 3.5 among non-Hispanic blacks and Mexican Americans, and among people with a BMI <25 or between 25 and 29 kg/m^2^. There was a significant interaction between the TG/HDL-C ratio and BMI for both sets of cutoff points, indicating that the association between TG/HDL-C ratio and hyperinsulinemia may differ by BMI categories in that this association was stronger among people with a lower category BMI than those with a higher BMI. However, there was no significant interaction between the TG/HDL-C ratio and race/ethnicity for hyperinsulinemia by using the new cutoff point.

**Table 3:** Prevalence, prevalence ratios, and 95% confidence intervals of hyperinsulinemia for the two TG/HDL-C ratio cutoff points by race and BMI categories, NHANES 1999–2002

	Cutoff point of 3.5 for the TG/HDL-C ratio					New cutoff point* for the TG/HDL-C ratio				
		
Characteristic	Prevalence, %		Prevalence ratio (≥ 3.5 vs. <3.5)			Prevalence, %		Prevalence ratio (≥ cutoff vs. <cutoff)		
				
	<3.5	≥ 3.5	PR	95% CI	<cutoff	≥ cutoff	PR	95% CI		
Total	15.65	48.07	2.16	1.74	2.08	12.95	44.71	2.23	1.83	2.72
Race										
Non-Hispanic White	13.0	45.6	2.3	1.7	3.1	11.3	41.9	2.3	1.8	3.0
Non-Hispanic Black	26.8	67.6	1.9	1.5	2.5	18.8	56.4	2.1	1.5	2.9
Mexican American	26.4	61.7	1.8	1.5	2.2	23.7	59.4	2.0	1.6	2.5
Interaction between race and TG/HDL-C ratio			χ^2 ^(2) = 5.05, P = 0.08					χ^2 ^(2) = 2.37, P = 0.31		
										
BMI, kg/m^2^										
< 25	3.4	14.3	3.3	1.4	7.4	2.8	13.7	3.8	1.6	8.8
25 to 29	13.3	38.2	2.9	2.0	4.2	10.5	35.8	3.4	2.4	5.0
≥ 30	46.1	71.4	1.5	1.3	1.8	43.6	68.0	1.5	1.3	1.7
Interaction between BMI and TG/HDL-C ratio			χ^2 ^(2) = 19.89, P < 0.0001					χ^2 ^(2) = 38.27, P < 0.0001		

* The new cutoff point of TG/HDL-C ratio is 3.0 in mg/dL unit (1.2 in mmol/L unit) for non-Hispanic whites and Mexican Americans and 2.0 in mg/dL unit (0.9 in mmol/L unit) for non-Hispanic blacks.

## Discussion

Using a large and nationally representative sample, we found that the TG/HDL-C ratio was significantly associated with FSI among nondiabetic adults of three major racial/ethnic subpopulations in the United States: non-Hispanic whites, non-Hispanic blacks, and Mexican Americans. We also detected racial/ethnic differences in predicting hyperinsulinemia, as both non-Hispanic whites and Mexican Americans had a larger optimal cutoff point of the TG/HDL-C ratio for the determination of hyperinsulinemia than did non-Hispanic blacks.

The association of insulin resistance with TG/HDL-C has been reported in overweight and obese whites [9,10]. Because the clinical samples that were used in these studies consisted primarily of white participants, the generalizability of this association has not been established. A recent study found that triglyceride concentration and the TG/HDL-C ratio were not significantly associated with insulin resistance (as determined from the insulin sensitivity index) in 125 African American participants [15]; this failure to find an association between the TG/HDL-C ratio and insulin resistance in blacks was perhaps attributable to the small sample and thus a limited power to detect associations. Our results, however, provide evidence that the TG/HDL-C ratio is associated with FSI in nondiabetic adults regardless of race/ethnicity.

Racial/ethnic differences in triglyceride concentrations, HDL-C values, and the FSI have been widely reported among adults and adolescents in previous studies [14,16-19]. In the present study, non-Hispanic blacks had lower triglyceride concentrations than non-Hispanic whites or Mexican Americans among both men and women; for HDL-C concentrations among males the values were highest among non-Hispanic blacks, but among women no real difference was seen in this value between blacks and whites. In both sexes the TG/HDL-C ratio was lower in blacks than in whites or Mexican Americans. In addition, a significant association of the TG/HDL-C ratio with insulin resistance (as measured by HOMA) was stronger among non-Africans than Africans [14], and increased triglyceride concentrations were associated with hepatic insulin resistance in white but not black adolescents [16]. Lipoprotein lipase activity has been found to be higher in blacks than in whites, and this may lead to a lower triglyceride concentration in blacks [29]. An elevated triglyceride concentration commonly causes low HDL-C values or regulates HDL-C remodeling [30-32], and thus these two clinically useful measures may be, at least in part, causally linked. In light of racial/ethnic differences in triglyceride concentrations, HDL-C, and the TG/HDL-C ratio, different cutoff points may be needed to define dyslipidemia. In fact, use of a single cutoff value of ≥ 150 mg/dl (or 1.69 mmol/L) for triglycerides as criteria to classify people with dyslipidemia have led to underestimation of the prevalence of the metabolic syndrome in blacks as compared with whites, particularly in men [33,34].

A TG/HDL-C ratio ≥ 3.5 has been proposed as a cutoff value to predict the presence of small-density LDL phenotype; McLaughlin and colleagues found that this cutoff had high sensitivity (79%) and specificity (85%) in the white population [10]. These authors also found, however, that this cutoff value had only moderate ability to predict insulin resistance (sensitivity 47%, specificity 88%) or the metabolic syndrome (sensitivity 46%, specificity 92%) [10]. We found, however, that the magnitude of the association of hyperinsulinemia with an elevated TG/HDL-C ratio was different when using the single cutoff point of 3.5, suggesting that a single cutoff point may not be applicable across diverse populations. In fact, our study showed, for the first time, that the optimal cutoff point of the TG/HDL-C ratio for prediction of hyperinsulinemia was 3.0 (in mg/dL unit) for non-Hispanic whites and Mexican Americans and 2.0 (in mg/dL unit) for non-Hispanic blacks, with reasonable sensitivity and specificity. Applying these race/ethnicity-specific cutoff points yielded similar magnitude of strength in the association across racial/ethnic subgroups.

It is of great interest that the association of the TG/HDL-C ratio with hyperinsulinemia was stronger among people with a BMI <25 kg/m^2 ^than those with a BMI ≥ 30 kg/m^2^. As shown in a previous study, about 16% people with normal weight (BMI < 25 kg/m^2^) was identified to be insulin resistant [35]; thus search for clinically simple and useful biomarkers to detect insulin resistance among people with normal weight is necessary. Although BMI, a less expensive and convenient measure, may serve as an indicator for insulin resistance among people with excessive weight, an elevation in the TG/HDL-C ratio could be a novel marker for hyperinsulinemia among people with normal weight in routine clinical practice.

Our study has several strengths that deserve attention. First, the generalizability of our results has been enhanced by the use of a nationally representative sample. Second, the large sample for each of the three racial/ethnic groups enables us to empirically test differences between the groups in the associations of the TG/HDL-C ratio with FSI. A limitation stems from the use of FSI as a surrogate measure of insulin resistance in our data, but previous studies have shown that fasting insulin is a valid and reliable surrogate measure of insulin resistance [13]. Furthermore, although a direct measure would theoretically be preferable, the lack of standardized insulin assay [36] greatly limits the clinical utility of that approach for identifying insulin-resistant persons. Without a standard insulin assay, no absolute value can be developed to predict the presence of insulin resistance across laboratories or clinical settings. Thus, if one wants to use fasting insulin to assess the state of insulin resistance, it is necessary to develop different cut-points for every assay. In contrast, triglyceride and HDL cholesterol concentrations are standardized and readily available measures in routine clinical practice. Consequently, it seems more clinically effective to use relatively simple metabolic markers such as the TG/HDL-C concentration ratio in the effort to identify apparently healthy people at increased risk of developing a variety of adverse clinical outcomes.

In conclusion, our findings have clinical and public health implications. In clinical settings, the TG/HDL-C ratio could be used as an indicator of insulin resistance across racial/ethnic subpopulations. In large health surveys, it could be used to monitor trends in cardiovascular health in diverse populations. Our results add further support to the notion that the TG/HDL-C ratio may be a clinically simple and useful indicator for insulin resistance among nondiabetic adults regardless of race/ethnicity. Even so, racial/ethnic-specific cutoff points of the TG/HDL-C ratio for the determination of insulin resistance or the risk of type 2 diabetes or cardiovascular diseases may be needed. Future research is warranted to assess the predictive power of the TG/HDL-C ratio for the metabolic syndrome, type 2 diabetes, or cardiovascular disease.

## Competing interests

The author(s) declare that they have no competing interests.

## Authors' contributions

CL, ESF, and YXM conceived the study. CL performed the statistical analysis and prepared the initial draft of the article. ESF, YXM, AHM, and GMR critically revised the manuscript for important intellectual content. All authors read and approved the final version of the manuscript.
